# Contact-dependent interactions between *Cutibacterium acnes* and *Staphylococcus lugdunensis* promote biofilm matrix remodeling

**DOI:** 10.21203/rs.3.rs-11542571/v1

**Published:** 2026-11-01

**Authors:** Bren Den Ng, Juliana S. Reviello, Rayssa L. Snider, Marcos Filipe Muniz Faria, Jessica F. S. Lopes, Eliane O. Ferreira, Leandro A. Lobo, Rosana B. R. Ferreira

**Affiliations:** University of Kansas; Universidade Federal do Rio de Janeiro; University of Kansas; Universidade Federal do Rio de Janeiro; Universidade Federal do Rio de Janeiro; Universidade Federal do Rio de Janeiro; Universidade Federal do Rio de Janeiro; University of Kansas

**Keywords:** Skin microbiota, Biofilm, polymicrobial infections, prosthetic joint infections, ica operon, polysaccharide intercellular adhesin

## Abstract

Prosthetic joint infections (PJIs) pose a substantial burden to patients and healthcare systems. These infections are often persistent and frequently require revision procedures, increasing the risk of surgical failure and mortality. A major virulence determinant underlying their persistence is biofilm formation, which protects bacteria from antibiotic treatment and host immune defenses. Understanding the mechanisms that promote biofilm formation is therefore critical for improving PJI management. *Staphylococcus* spp. and *Cutibacterium acnes* are common skin commensals that are frequently co-isolated from chronic prosthetic device infections. We hypothesized that these organisms interact synergistically to enhance the persistence of polymicrobial infections. We found that *C. acnes* increases dual-species biofilm formation with *Staphylococcus lugdunensis* and *Staphylococcus hominis* relative to monocultures. This phenotype was specific to these species combinations and was widespread among the strains tested. To investigate the underlying mechanism, we focused on the interaction between *C. acnes* and *S. lugdunensis*. Enhanced biofilm formation required both species to be metabolically active and in direct physical contact. Notably, co-culture shifted *S. lugdunensis* biofilms from their typical proteinaceous matrix to a polysaccharide intercellular adhesin (PIA)-rich matrix. Consistent with this transition, co-culture with *C. acnes* significantly increased expression of *icaA*, a gene involved in PIA biosynthesis, while having no effect on expression of *atlL* and *mgtE*, genes associated with the proteinaceous matrix of *S. lugdunensis*. Together, these findings reveal a novel contact-dependent interspecies interaction in which *C. acnes* promotes a switch to PIA-mediated biofilm formation in *S. lugdunensis*, a mechanism that may enhance persistence on medical devices and contribute to chronic polymicrobial infections.

## Introduction

Prosthetic joint infections (PJIs) are among the most serious complications following joint arthroplasty and represent a substantial clinical and economic burden^1^. As the number of arthroplasty procedures continues to rise worldwide, the incidence and healthcare costs associated with PJIs are expected to increase accordingly^22^. Although PJIs occur in only a small proportion of implant procedures (ranging from 0.5–2.3%), they frequently require prolonged antibiotic therapy and revision surgery and are associated with significant morbidity, mortality, and healthcare expenditures. Furthermore, the true burden of PJIs is likely underestimated because repeated hospitalizations, long-term follow-up care, and indirect costs are often not fully captured^3^.

While most studies of PJIs have focused on monomicrobial infections, polymicrobial infections are generally associated with poorer clinical outcomes, increased treatment failure, and prolonged disease persistence^4^. These infections frequently exhibit distinct microbial compositions and pathogenic characteristics compared with monomicrobial infections, suggesting that interactions between co-infecting microorganisms may contribute to disease progression, persistence and severity^4,5^. Despite their clinical importance, the mechanisms through which bacterial species interact within prosthetic-associated infections remain poorly understood.

Among the microorganisms commonly isolated from PJIs, *Cutibacterium acnes* and coagulase-negative staphylococci (CoNS) are particularly prevalent^6,7^. *C. acnes*, a common anaerobic skin commensal, is the predominant pathogen associated with shoulder PJIs and is frequently recovered alongside CoNS in polymicrobial infections^8-10^. Despite their frequent co-isolation, little is known about how these organisms interact and whether such interactions influence biofilm development and infection persistence.

The ability to adhere to biotic surfaces, such as the skin and heart valves, or abiotic surfaces, including prosthetic devices, and subsequently form biofilms is a major virulence determinant of both *Staphylococcus* spp. and *C. acnes*^11,12^. Biofilm-associated bacteria account for a large proportion of chronic bacterial infections and are a leading cause of medical implant failure^13,14^. The biofilm matrix is a complex structure composed of proteins, polysaccharides, extracellular DNA, and lipids, and its composition varies considerably among bacterial species and strains^15^. By limiting antimicrobial penetration and protecting cells from host immune defenses, biofilms enable bacterial persistence and contribute to the chronicity and severity of implant-associated infections^16^.

Increasing evidence indicates that interactions within polymicrobial biofilms can profoundly alter bacterial physiology, antimicrobial tolerance, and virulence^12,17-19^. Interspecies interactions can promote biofilm maturation through metabolic cooperation, cell-cell signaling, or alterations in extracellular matrix production. For example, co-culture of *Staphylococcus aureus* and *Candida albicans* results in enhanced biofilm formation and increased antimicrobial tolerance compared with either species grown alone^20,21^. Similarly, interactions between *Pseudomonas aeruginosa* and *Staphylococcus aureus* can alter biofilm architecture, metabolic activity, and antibiotic susceptibility, contributing to chronic infection persistence^22,23^. In oral polymicrobial communities, cooperative interactions among streptococci and other oral bacteria facilitate biofilm development and colonization of host surfaces^24^. These observations highlight the capacity of microbial interactions to reshape biofilm behavior in ways that are not predicted from monoculture studies. However, little is known about how skin commensals commonly associated with PJIs influence one another's biofilm programs.

*Staphylococcus lugdunensis*, a member of the CoNS group and a normal constituent of the skin microbiota, has emerged as an important opportunistic pathogen^25,26^. Unlike many CoNS species, *S. lugdunensis* can cause highly invasive infections, including infective endocarditis, urinary tract infections, catheter-associated infections, and prosthetic joint infections^25^. Its ability to form biofilms likely contributes to its persistence during infection and reduced susceptibility to antimicrobial therapy^27,28^. Notably, although *S. lugdunensis* possesses the *icaADBC* operon, which mediates synthesis of polysaccharide intercellular adhesin (PIA) in several staphylococcal species, biofilms formed by clinical isolates are often predominantly proteinaceous^29,30^. This apparent discrepancy suggests that environmental signals or interactions with neighboring microorganisms may influence biofilm matrix composition in *S. lugdunensis*.

Despite the frequent co-isolation of *C. acnes* and CoNS from prosthetic-associated infections, it remains unclear whether *C. acnes* can modulate biofilm development by neighboring staphylococci. Understanding such interactions may provide important insight into how otherwise benign members of the skin microbiota cooperate to persist on implanted medical devices and contribute to chronic infection.

Here, we investigated whether interactions between *C. acnes* and *Staphylococcus* spp. influence biofilm formation in polymicrobial communities associated with prosthetic-device infections. We identified species-specific interactions in which *C. acnes* enhanced biofilm formation by *Staphylococcus lugdunensis* and *Staphylococcus hominis*. Furthermore, we demonstrate that the interaction with *S. lugdunensis* is contact-dependent and is associated with induction of the *ica* locus and a shift from a predominantly proteinaceous matrix to a polysaccharide intercellular adhesin (PIA)-rich biofilm state. These findings reveal a previously unrecognized mechanism through which a common skin commensal can reprogram staphylococcal biofilm matrix composition, providing new insight into microbial cooperation during prosthetic-associated infections.

## Materials and Methods

### Bacterial Strains and Growth Conditions

All bacterial strains were stored at − 80°C in tryptic soy broth (TSB) supplemented with 20% glycerol. *Cutibacterium acnes* strains were routinely cultured on blood agar plates for 72 h at 37°C under anaerobic conditions (80% N_2_, 10% O_2_ and 10% H_2_), whereas *Staphylococcus* spp. isolates were cultured on tryptic soy agar (TSA; Becton, Dickinson & Co., Sparks, MD, USA) plates for 24 h at 37°C under aerobic conditions. The bacterial strains used in this study are listed in Table S1.

#### Semiquantitative Analysis of Biofilm Formation

Biofilm formation was assessed using the polystyrene microtiter plate assay as previously described^31^, with minor modifications. Bacterial suspensions containing 3 x 10^8^ colony-forming units (CFU)/mL were prepared in TSB supplemented with 1% (w/v) glucose. Twenty microliters of each suspension were added to 180 μL of fresh TSB supplemented with 1% glucose in 96-well polystyrene plates (TPP-Techno Plastic Products, Trasadingen, Switzerland). For dual-species biofilms, 10 μL of each bacterial suspension was added to the same well, maintaining a total inoculum volume of 20 μL. Plates were incubated under anaerobic conditions at 37°C for either 24 h or 72 h. Following incubation, planktonic cultures were removed and the wells were washed three times with phosphate-buffered saline (PBS; Ph 7.2; Fisher Bioreagents, USA). Biofilms were fixed at 60°C for 1 h and stained with 200 μL of 0.1% safranin per well for 15 min at room temperature. Wells were subsequently rinsed twice with PBS, and 200 μL of 95% ethanol was added to solubilize the stain. After 30 min, absorbance was measured at 492 nm (OD492nm) using a microplate reader. Biofilm biomass was calculated by subtracting the mean absorbance of negative control wells containing sterile medium from the absorbance of experimental wells. Each experiment was performed in technical triplicate and repeated independently at least twice.

#### Analysis of Biofilm Formation on Titanium Discs

Monocultures of *C. acnes*, *S. lugdunensis*, and *S. hominis*, as well as cocultures of *C. acnes* with either *S. lugdunensis* or *S. hominis*, were prepared as described above and inoculated into 6-well plates (TPP) containing TSB supplemented with 1% glucose and sterile titanium discs (Sigma-Aldrich, St. Louis, MO, USA). Plates were incubated under anaerobic conditions at 37°C for 72 h. Biofilms formed on titanium discs were washed three times with sterile PBS and quantified using the safranin staining protocol described above. For scanning electron microscopy (SEM), discs were washed three times with PBS and fixed overnight at 4°C in Karnovsky's fixative (5% glutaraldehyde and 4% formaldehyde in 0.1 M sodium cacodylate buffer). Samples were subsequently washed three times with 0.2 M sodium cacodylate buffer for 15 min each and post-fixed in 1% osmium tetroxide prepared in 0.1 M sodium cacodylate buffer for 1 h. Discs were washed again and dehydrated through a graded ethanol series (30%, 50%, 70%, 90%, and 100%). Samples were dried using a CO_2_ critical-point dryer, mounted onto aluminum stubs using carbon double-sided adhesive tape, and sputter-coated with gold. Biofilms were visualized using a Quanta 250 FEI scanning electron microscope (Thermo Fisher Scientific, Waltham, MA, USA), and representative micrographs were acquired at 5,000×, 10,000×, and 40,000× magnification.

#### Effect of Secreted Molecules on Biofilm Development

Cell-free conditioned media from *C. acnes* and *S. lugdunensis* were prepared as described earlier^32-34^. Briefly, *S. lugdunensis* and *C. acnes* grown anaerobically for 48 h and 72 h, respectively, in TSB supplemented with 1% glucose were centrifuged, filtered (0.22-μm), and concentrated 20-fold using a SpeedVac. CFCM preparations were used at a final concentration of 2-fold. Plates were incubated anaerobically at 37°C for 72 h and biofilm formation assays were performed as described earlier and. Control conditioned medium was prepared from uninoculated culture medium.

#### Effect of Bacterial Viability on Dual-Species Biofilm Development

Bacterial suspensions were prepared as described above. Prior to inoculation, bacterial suspensions were exposed to UV irradiation for 1 h to generate nonviable cells while maintaining cellular integrity. UV-treated suspensions were then used in place of live bacteria in the dual-species biofilm assay, while live-cell cocultures were included as controls. To confirm cell death, 50 μL of each UV-treated suspension was plated onto TSA plates and incubated under the appropriate conditions. Biofilms were incubated under anaerobic conditions for 24 h and quantified as described above.

### Evaluation of Contact-Dependent Interactions During Dual-Species Biofilm Formation

Bacterial suspensions were prepared as described above. To evaluate the requirement for physical contact, dual-species biofilms were established using 24-well plates fitted with transwell inserts (0.4-μm pore size; Corning, USA). Briefly, 100 μL of *S. lugdunensis* suspension and 400 μL of TSB supplemented with 1% glucose were added to the lower chamber. The upper insert received 100 μL of *C. acnes* inoculum and 400 μL of TSB supplemented with 1% glucose. The reciprocal arrangement was also evaluated. Monocultures and cocultures established without transwell inserts served as controls. Plates were incubated under anaerobic conditions for 24 h, after which biofilm biomass formed in the lower chamber was quantified as described above.

#### Evaluation of the Contribution of Matrix Components to Biofilm Formation

Bacterial suspensions were prepared as described above. To evaluate the contribution of different matrix components to biofilm formation, DNase I (10 μg/mL), proteinase K (1.25 μg/mL), or sodium periodate (0.625 mM) was added to the media at the time of inoculation. Plates were incubated under anaerobic conditions for 24 h, and biofilm biomass was quantified as described above.

#### Enzymatic and Chemical Disruption of Established Biofilms

The relative contribution of polysaccharides, eDNA and proteins to the biofilm matrix of established biofilms of monocultures and co-cultures was evaluated as previously described^30^ with modifications. Briefly, planktonic cells were carefully removed from 24-h biofilms inoculated as described earlier, the biofilm mass was then fixed for 1 h at 60 °C, and incubated with either PBS (control), DNase I (100 μg/mL), proteinase K (20 mg/mL), or sodium periodate (10 mM) for 1 h at 37°C. Following treatment, biofilms were washed once with PBS, and residual biofilm biomass was quantified using the safranin staining assay described above.

### Determination of the Relative Contribution of Each Species to Enhanced Biofilm Formation

Bacterial suspensions were prepared as described above. Dual-species inocula were generated with varying proportions of each species (50%, 25%, 10%, 1%, 0.1%, 0.01%, 0.001%, and 0.0001%), with the second species comprising the remainder of the total inoculum. Plates were incubated under anaerobic conditions for 24 h, and biofilm formation was quantified as described above.

### Evaluation of the Effect of Colonization Order on Enhanced Biofilm Formation

To determine whether the timing of colonization influenced biofilm formation, 10 μL of either *C. acnes* or *S. lugdunensis* suspension was inoculated into wells containing 190 μL of TSB supplemented with 1% glucose. Plates were incubated under anaerobic conditions for 12 h. Following incubation, culture densities were measured at OD600, and a suspension of the second bacterial species was adjusted to match the optical density of the established culture. The second species was then added to generate dual-species biofilms. Plates were incubated for an additional 12 h under anaerobic conditions before biofilm biomass was quantified as described above.

### Assessment of Extracellular Polysaccharide Production by Epifluorescence Microscopy

Biofilms formed by *C. acnes*, *S. lugdunensis*, and their coculture were analyzed by epifluorescence microscopy as previously described with modifications^35^. Briefly, mono-species and dual-species biofilms were established in 96-well glass-bottom plates (Cellvis, Mountain View, CA, USA) and incubated for 24 h at 37°C under anaerobic conditions. Following incubation, culture medium was removed and the biofilms were washed twice with PBS. Biofilms were stained either with the LIVE/DEAD BacLight Bacterial Viability Kit (Thermo Fisher Scientific, Waltham, MA, USA), according to the manufacturer's instructions, or with wheat germ agglutinin (WGA)-Texas Red (Thermo Fisher Scientific) to visualize extracellular polysaccharides. Samples were incubated for 45 min at room temperature under gentle agitation (150 rpm) and protected from light. Wells were then washed twice with PBS prior to imaging on an Olympus IX-81 inverted microscope. Images were processed using ImageJ software (National Institutes of Health, Bethesda, MD, USA), and fluorescence intensity was quantified and normalized.

#### Extracellular polysaccharide quantification

Extracellular polysaccharides were quantified using the phenol-sulfuric acid method as previously described, with minor modifications^36,37^. Briefly, solubilized biofilm matrix samples from monocultures and co-cultures were mixed with 80% phenol and concentrated sulfuric acid and incubated according to the standard protocol. Total polysaccharide content was determined by measuring absorbance at 490 nm using an Infinite F50 microplate reader (Tecan, Männedorf, Switzerland). Polysaccharide concentrations were calculated from a standard curve generated using serial dilutions of glucose (1 mg/mL).

### RNA Extraction and Quantitative PCR (qPCR)

Mono-species and dual-species biofilms were established in 6-well polystyrene plates (TPP) with a final volume of 2 mL per well and incubated under anaerobic conditions for 24 h. RNA extraction was adapted from Glatthardt et al., with modifications^33^. Following incubation, biofilms were washed three times with 2 mL PBS to remove planktonic cells. Biofilms were then resuspended in 2 mL RNAprotect reagent (Qiagen, Hilden, Germany) and sonicated for 5 min at high intensity using a Branson CPX2800H Digital Ultrasonic Cleaner. Biofilms were subsequently scraped using sterile pipette tips, and recovered cells were pelleted by centrifugation. Cell pellets were treated with a lysis solution containing lysozyme (40 mg/mL), lysostaphin (40 μg/mL), and proteinase K (6 mg/mL). Total RNA was isolated using the SV Total RNA Isolation System (Promega, Madison, WI, USA) according to the manufacturer's instructions. Residual genomic DNA was removed using TURBO DNase (Thermo Fisher Scientific). Complementary DNA (cDNA) was synthesized using the GoScript Reverse Transcription System (Promega). Quantitative PCR was performed on a QuantStudio 3 Real-Time PCR System (Applied Biosystems, Waltham, MA, USA) using PowerTrack SYBR Green Master Mix (Applied Biosystems). Gene expression levels were normalized to the housekeeping gene *recA*, which encodes a DNA recombinase involved in homologous recombination and DNA repair. Relative gene expression was calculated using the 2^-ΔΔCt method. Primer sequences are listed in Table S2.

### Statistical Analysis

Statistical analyses were performed using GraphPad Prism version 10 (GraphPad Software, San Diego, CA, USA). Data are presented as mean ± standard deviation (SD). Experiments were performed in triplicate and repeated at least twice. Comparisons between two groups were performed using Student's *t*-test, whereas comparisons among multiple groups were performed using one-way analysis of variance (ANOVA) followed by Tukey's multiple-comparison test. A *P* value of < 0.05 was considered statistically significant.

## Results

### C. acnes selectively enhances biofilm formation by S. lugdunensis and S. hominis

To determine whether *Cutibacterium acnes* influences biofilm formation by skin-associated staphylococci, we compared biofilm formation in mono- and dual-species cultures. Under the conditions tested, *C. acnes* formed little detectable biofilm in monoculture, whereas most *Staphylococcus* species produced measurable biofilms (Fig. 1A). Dual-species cultures of *C. acnes* with *S. epidermidis*, *S. aureus*, *S. haemolyticus*, *S. capitis*, or *S. saprophyticus* did not exhibit significant differences in biofilm biomass relative to the corresponding monocultures. In contrast, co-culture of *C. acnes* with either *S. lugdunensis* or *S. hominis* resulted in a marked increase in biofilm biomass (Fig. 1A). Evaluation of multiple strains from each species revealed a consistent phenotype, indicating that the interaction was not strain specific (**Figure S1**).

To determine whether this phenotype was relevant to prosthetic surfaces, biofilm formation was assessed on titanium discs. Although *C. acnes* produced greater biofilm biomass on titanium than on polystyrene, co-culture with either *S. lugdunensis* or *S. hominis* further enhanced biofilm formation compared to the corresponding monocultures (Fig. 1B-C). Scanning electron microscopy confirmed these findings. Monocultures of *C. acnes* and *S. hominis* displayed sparse bacterial aggregates on the titanium surface, whereas *S. lugdunensis* formed larger but still discontinuous clusters. In contrast, dual-species biofilms exhibited extensive surface coverage and a denser biofilm architecture (Fig. 1D-E).

Together, these findings demonstrate that *C. acnes* selectively enhances biofilm formation by *S. lugdunensis* and *S. hominis* on both polystyrene and clinically relevant titanium surfaces.

### Enhanced biofilm formation requires viable cells and direct physical contact

Because the strongest phenotype was observed with *S. lugdunensis*, subsequent experiments focused on this interaction. To determine whether enhanced biofilm formation required bacterial viability, one species was rendered nonviable by UV treatment prior to co-culture. Enhanced biofilm formation was observed only when both species were viable, indicating that metabolically active cells are required for the interaction (Fig. 2A).

We next tested whether direct physical contact was necessary for biofilm enhancement using a transwell system that prevented cell-cell contact while permitting diffusion of soluble molecules. Physical separation of *C. acnes* and *S. lugdunensis* completely abolished the enhanced biofilm phenotype (Fig. 2B). Biofilms of *C. acnes* and *S. lugdunensis* containing cell-free conditioned media (CFCM) obtained from either *S. lugdunensis* or *C. acnes* were evaluated, revealed that molecules produced by one of the species do not increase the biofilm of the other under the tested conditions (**Figure S2**). In fact, addition of *C. acnes* CFCM significantly inhibited *S. lugdunensis* biofilm formation. These results indicate that increased biofilm formation requires both viable bacteria and direct interaction between the two species.

#### Biofilm enhancement is highly dependent on the presence of S. lugdunensis

To determine the relative contribution of each organism to the enhanced biofilm phenotype, co-cultures were established using varying proportions of each species. Reducing the proportion of *S. lugdunensis* had little effect on biofilm formation until the species represented less than 0.01% of the total population (Fig. 3A). In contrast, at least 10% *C. acnes* was required to maintain the enhanced biofilm phenotype (Fig. 3B).

We next examined whether the timing of colonization influenced biofilm development. When *S. lugdunensis* was established for 12 h prior to the introduction of *C. acnes*, biofilm biomass remained comparable to simultaneously inoculated co-cultures. However, when *C. acnes* was established first and *S. lugdunensis* was added 12 h later, enhanced biofilm formation was no longer observed (Fig. 3C). Given the substantially slower growth rate of *C. acnes* compared to *S. lugdunensis*, these findings suggest that *S. lugdunensis* is the principal contributor to biofilm accumulation, whereas *C. acnes* functions as a stimulatory partner that induces this phenotype.

### Enhanced biofilm formation is associated with polysaccharide production

To investigate whether biofilm enhancement was affected by degradation of extracellular DNA (eDNA), proteins, or polysaccharides, common components of biofilm matrices, biofilms were formed in the presence of DNase I, proteinase K, or sodium periodate, respectively. DNase I and proteinase K significantly reduced the ability of *S. lugdunensis* monocultures and co-cultures to form biofilms, indicating that eDNA and protein components contribute to biofilm formation on both conditions (Fig. 4A-B).

In contrast, addition of sodium periodate, which degrades extracellular polysaccharides, had markedly different effects on monocultures and co-cultures. Whereas degradation of polysaccharides did not significantly affect biofilm formation by *S. lugdunensis* alone, it reduced dual-species biofilms to levels comparable to those observed in the monoculture (Fig. 4C).

To determine what matrix components were present in established mono species and dual-species biofilms, we treated 24-h biofilms with either DNaseI, proteinase K or sodium periodate (Fig. 4D). Our data indicated that eDNA and proteins are required for both monocultures and co-cultures. Degradation of polysaccharides, however, only significantly affected co-culture biofilms. These findings indicate that polysaccharides are specifically required for the enhanced biofilm phenotype and suggest that co-culture with *C. acnes* promotes a shift in matrix composition toward greater polysaccharide dependence.

#### Co-culture with C. acnes promotes extracellular polysaccharide production and induces icaA expression in S. lugdunensis

To directly examine extracellular polysaccharide production, mono- and dual-species biofilms were stained with wheat germ agglutinin (WGA), a lectin that binds extracellular polysaccharides. Fluorescence microscopy revealed substantially greater WGA staining in dual-species biofilms than in *S. lugdunensis* monocultures, indicating increased accumulation of extracellular polysaccharides during coculture (Fig. 5A-B). Furthermore, quantitative analysis of polysaccharides extracted from the biofilm matrix confirmed the presence of significant higher levels of extracellular polysaccharides in co-cultures than in monocultures of either species (Fig. 5C).

To determine whether this phenotypic transition was associated with altered gene expression, we quantified expression of genes involved in *S. lugdunensis* biofilm formation. Co-culture with *C. acnes* induced approximately 9-fold higher expression of *icaA*, a key gene involved in polysaccharide intercellular adhesin (PIA) biosynthesis, compared with monoculture biofilms (Fig. 5D). In contrast, expression of *mgtE* and *atlL*, genes associated with protein-mediated biofilm formation in *S. lugdunensis*, was not significantly altered.

Collectively, these findings support a model in which direct interaction between *C. acnes* and *S. lugdunensis* induces *icaA* expression and promotes a transition from a predominantly proteinaceous biofilm matrix to a PIA-rich matrix. This matrix remodeling is associated with enhanced biofilm accumulation and may represent a mechanism contributing to persistence during polymicrobial prosthetic-associated infections.

## Discussion

Microbial interactions play a critical role in shaping community structure, persistence, and virulence^17,38-43^. Within polymicrobial communities, neighboring organisms can engage in cooperative, competitive, or neutral interactions that profoundly influence microbial physiology and adaptation to specific host environments. Recent studies have highlighted the importance of pathogen-pathogen interactions in determining infection outcomes, particularly in chronic biofilm-associated diseases^24,44^. Examples include the cooperative co-aggregation networks observed within the oral microbiome and the complex interactions between *Staphylococcus aureus* and *Pseudomonas aeruginosa* in cystic fibrosis and chronic wound infections^45-48^. Collectively, these observations emphasize that the behavior of a microorganism within a community can differ substantially from that observed in monoculture.

In the present study, we investigated interactions between *Cutibacterium acnes* and members of the genus *Staphylococcus* that are frequently recovered from prosthetic joint infections^49,50^. We demonstrate that *C. acnes* selectively enhances biofilm formation by *S. lugdunensis* and *S. hominis*, whereas no significant effect was observed for several other staphylococcal species. Importantly, this phenotype was consistent across multiple strains of all three species, suggesting that the interaction is broadly conserved rather than strain specific. The observation that only two staphylococcal species responded to *C. acnes* indicates that the phenotype is unlikely to result from a nonspecific environmental alteration, such as nutrient consumption or metabolic byproducts. Instead, these findings suggest the existence of species-specific mechanisms that enable selected members of the skin microbiota to cooperate during biofilm development.

Several studies have reported interactions between *C. acnes* and staphylococci that influence biofilm formation. Molecules secreted by *C. acnes* have been shown by us and others to inhibit biofilm formation by *S. epidermidis*^51^, *S. lugdunensis* and *S. hominis*^32,34^. In contrast, staphylococcal species, including *S. epidermidis*, *S. hominis*, *S. capitis* and *S. aureus* have been reported to indirectly promote *C. acnes* biofilm formation through the creation of localized anaerobic environments^52^. Furthermore, *C. acnes* has also been shown to prolong *S. aureus* survival within dual-species biofilms under anaerobic conditions^53^. However, whether these effects require direct physical interaction between the species or involve active remodeling of extracellular biofilm matrix has remained largely unexplored. Our findings identify a distinct type of interaction between *C. acnes* and *S. lugdunensis* in which direct association between the organisms promotes biofilm development and alters biofilm matrix composition Notably, the phenotype was species specific, being observed only in *S. lugdunensis* and *S. hominis* among the staphylococcal species tested. Together, these observations highlight the importance of environmental and ecological context in shaping microbial interactions. Depending on the surrounding conditions, the same organisms may engage in either antagonistic or cooperative behaviors, likely reflecting adaptation to different niches ranging from healthy skin to implanted medical devices and chronic infections.

Our study revealed that enhanced biofilm formation in *C. acnes* and *S. lugdunensis* co-cultures required both viable bacteria and direct physical contact. UV-killed cells were unable to support the phenotype, and physical separation of the species using transwell membranes completely abolished biofilm enhancement. Moreover, molecules produced by one species did not increase biofilm formation by the other. Together, these observations strongly suggest that the interaction is not mediated by diffusible molecules. Rather, direct contact between the two live species is necessary to initiate the response, arguing against one species being solely a scaffold for the other. Contact-dependent interactions are increasingly recognized as important mechanisms of communication among bacteria, allowing neighboring cells to exchange information, cooperate, compete, and respond to environmental stimuli^41,54-56^. Given the timing of biofilm production and the different doubling times of *S. lugdunensis* and *C. acnes*, the requirement for physical contact observed here indicates that *C. acnes* may function as a biological cue that alters the biofilm developmental program of *S. lugdunensis*.

Ours findings revealed that interaction with *C. acnes* induces a shift in the biofilm matrix composition of *S. lugdunensis*. Previous studies have reported that biofilms formed by clinical isolates of *S. lugdunensis* are predominantly proteinaceous and rely heavily on extracellular proteins and eDNA^30,57,58^. Consistent with these reports, biofilm formation by *S. lugdunensis* monocultures was highly sensitive to proteinase K treatment and largely unaffected by degradation of polysaccharides. Surprisingly, co-culture with *C. acnes* generated a biofilm state that was strongly dependent on polysaccharides. Sodium periodate treatment abolished the enhanced biofilm phenotype, and microscopy demonstrated the accumulation of extracellular polysaccharides specifically within dual-species biofilms. These findings indicate that matrix composition in *S. lugdunensis* is not fixed but can be actively remodeled in response to neighboring microorganisms.

This matrix remodeling was accompanied by a significant increase in *icaA* expression. Although the *icaADBC* locus is broadly conserved among *S. lugdunensis* strains, its role in biofilm formation has remained unclear because most clinical isolates do not appear to rely heavily on polysaccharide production for biofilm development like in other *Staphylococcus* species^59-61^. Our results suggest that the *ica* locus remains fully functional and can be activated under specific environmental conditions. Direct interaction with *C. acnes* induced approximately nine-fold greater *icaA* expression while having little effect on genes associated with protein-mediated biofilm formation. Together, these findings support a model in which *C. acnes* triggers a developmental transition in *S. lugdunensis* from a predominantly protein-based biofilm program to a polysaccharide intercellular adhesin (PIA)-rich state. More broadly, these observations support the emerging concept that biofilm matrix composition is a dynamic and environmentally responsive trait rather than a fixed species characteristic.

The molecular mechanisms responsible for this transition remain unknown. The absence of a requirement for diffusible factors and the strict dependence on direct contact suggest involvement of cell surface structures or contact-dependent signaling pathways. One possibility is that adhesins expressed by either species facilitate intimate physical interactions that trigger downstream regulatory responses. Another possibility is that cell envelope-associated polysaccharides participate directly in interspecies recognition. Consistent with this hypothesis, sodium periodate specifically disrupted the cooperative component of the biofilm phenotype, implicating polysaccharide-containing structures in the interaction. *C. acnes* has recently been shown to produce poly-β-(1→6)-N-acetyl-D-glucosamine (PNAG), a cell-surface polysaccharide involved in biofilm formation, whereas PIA produced by staphylococci is a structurally related polymer that mediates cell-cell adhesion^62^. Whether these polysaccharides contribute directly to the interaction described here remains unknown, but they represent potential candidates for future investigation.

The requirement for direct contact also raises the possibility that specialized signaling systems participate in this response. Type VII secretion systems (T7SS) have emerged as important mediators of contact-dependent interactions among Gram-positive bacteria and can influence interbacterial competition, host colonization, and biofilm formation^63^. While *C. acnes* does not appear to encode a canonical T7SS, *S. lugdunensis* contains genes predicted to form part of this secretion machinery^64^. Whether T7SS-associated proteins contribute to the cooperative interaction observed here remains to be determined. Future studies employing transcriptomics, targeted mutagenesis, and genetic screening approaches will be necessary to identify the signals and regulatory pathways linking interspecies contact to *ica* induction.

The ecological and clinical implications of this interaction may be extensive. Both *C. acnes* and *S. lugdunensis* are generally regarded as commensal members of the skin microbiota, yet both are increasingly recognized as opportunistic pathogens associated with PJIs and other device-related infections^4,25,27,65-67^. Our findings suggest that these organisms may exhibit behaviors in the context of implanted biomaterials that differ markedly from those observed on healthy skin. By promoting a PIA-rich biofilm state, *C. acnes* may enhance the ability of *S. lugdunensis* to establish persistent surface-associated communities on implanted devices. While the impact of this transition on antibiotic tolerance and host immune evasion remains to be determined, PIA-mediated biofilms in other staphylococci are known to contribute to biofilm stability and persistence^68-70^ Consequently, interspecies cooperation may represent an underappreciated contributor to chronic implant-associated infections.

The molecular mechanism by which *C. acnes* interaction regulates *icaA* expression in *S. lugdunensis* remains unknown. The genome of *S. lugdunensis* lacks the *icaR* gene, which encodes a repressor protein and is responsible for modulating *ica* expression in other *ica*-dependent biofilm forming strains of *Staphylococcus*^59^. It has also been shown that the *ica* operon may be regulated by factors outside of *icaR*, with the discovery of one other locus *rob* which helps regulate *ica* expression in *S. aureus*^71^. However, presence of this locus in *S. lugdunensis* is unclear. Future studies should focus on determining the signaling pathway responsible for *icaA* expression in *S. lugdunensis* and determine whether the same molecular mechanisms are involved on *C. acnes* interaction with *S. hominis*. Furthermore, studies employing implant-associated infection models are still required to determine the contribution of this interaction to persistence *in vivo*.

In conclusion, we identified a previously unrecognized interaction between *C. acnes* and *S. lugdunensis* that enhances biofilm formation through a contact-dependent mechanism. We show that co-culture with *C. acnes* induces *icaA* expression and drives a transition from a predominantly proteinaceous biofilm matrix to a PIA-rich state in *S. lugdunensis*. These findings reveal that interactions among skin commensals can actively reprogram biofilm development and matrix composition, providing a potential explanation for the persistence of polymicrobial communities on implanted medical devices. More broadly, our study highlights the importance of considering pathogen-pathogen interactions during biofilm-associated infections and suggests that disrupting cooperative microbial behaviors may represent a promising strategy for the prevention and treatment of chronic prosthetic joint infections.

## Supplementary Material

This is a list of supplementary files associated with this preprint. Click to download.

• Ngetal2026.SupplementaryInformation.docx

## Figures and Tables

**Figure 1:**
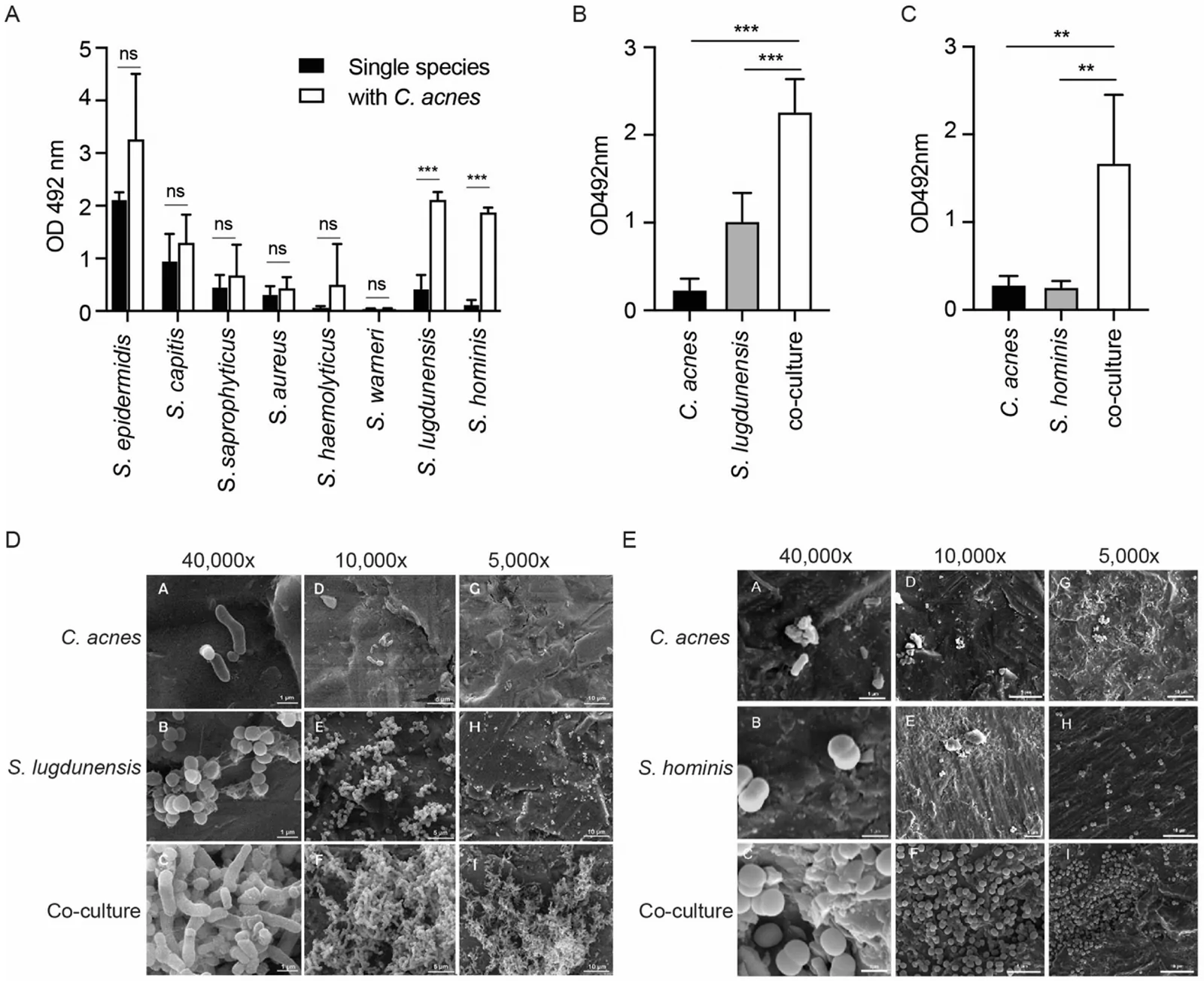
Co-culture of *C. acnes* with either *S. lugdunensis* or *S. hominis* increases total biofilm formation. A. Biofilm formation of *S. epidermidis* (ATCC 35984), *S. capitis* (95s), *S. saprophyticus* (MD03), *S. aureus* (112), *S. haemolyticus*(1046), *S. warneri* (1047), *S. lugdunensis* (546), and *S. hominis* (1001) with and without the presence of *C. acnes* (LCP) after 72 h of incubation. B. Biofilm formation of *C. acnes, S. lugdunensis* and co-cultures on titanium discs after 72 h of incubation. C. Biofilm formation of *C. acnes, S. hominis* and co-cultures on titanium discs after 72 h of incubation. D. Representative images of scanning electron microscopy of *C. acnes, S. lugdunensis* and co-culture formed on titanium discs after 72 h of incubation. E. Representative images of scanning electron microscopy of *C. acnes, S. hominis* and co-culture formed on titanium discs after 72 h of incubation. Mean values of 2 independent experiments are shown, and error bars represent the SD. Statistical analyses were performed with Multiple unpaired t- test (A), the Mann-Whitney test (B and C). **P<0.01; ***P<0.001; ns: not significant.

**Figure 2:**
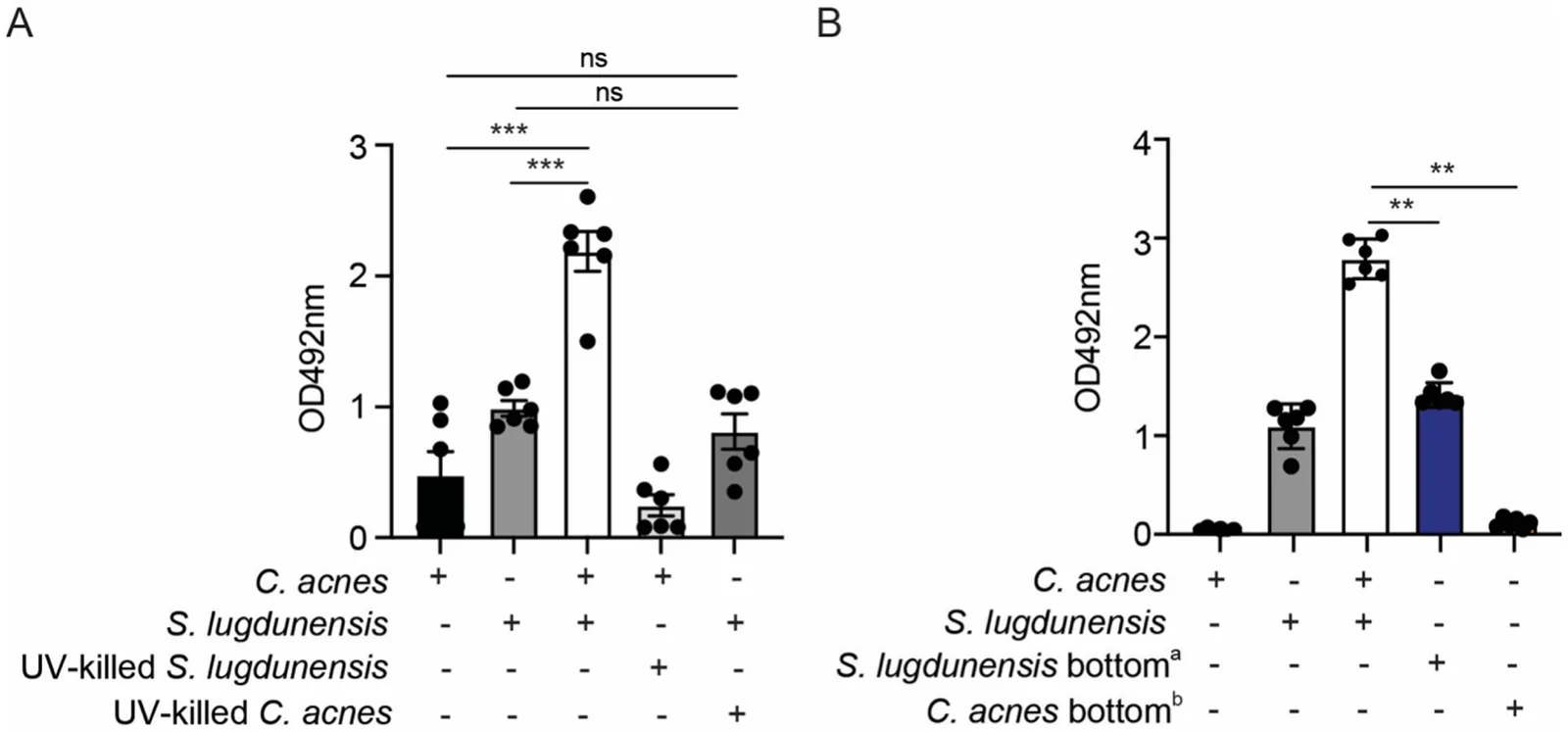
*C. acnes* and *S. lugdunensis* need to be metabolically active and in physical contact to increase biofilm formation. A. Biofilm formation of co-cultures containing either UV-killed *C. acnes* or *S. lugdunensis* after 24h of incubation. B. Biofilm formation of either *C. acnes* or *S. lugdunensis* in the presence of the other species separated by a transwell filter after 24h of incubation. ^a^: *S. lugdunensis* biofilm in the presence of physically separated *C. acnes*; ^b^: *C. acnes* biofilm in the presence of physically separated *S. lugdunensis*. Dots represent individual values obtained from two independent experiments and bars represent SEM. Statistical analyses were performed using Welch’s t-test. ** *P*<0.01 *** *P*<0.001; ns: not significant.

**Figure 3:**
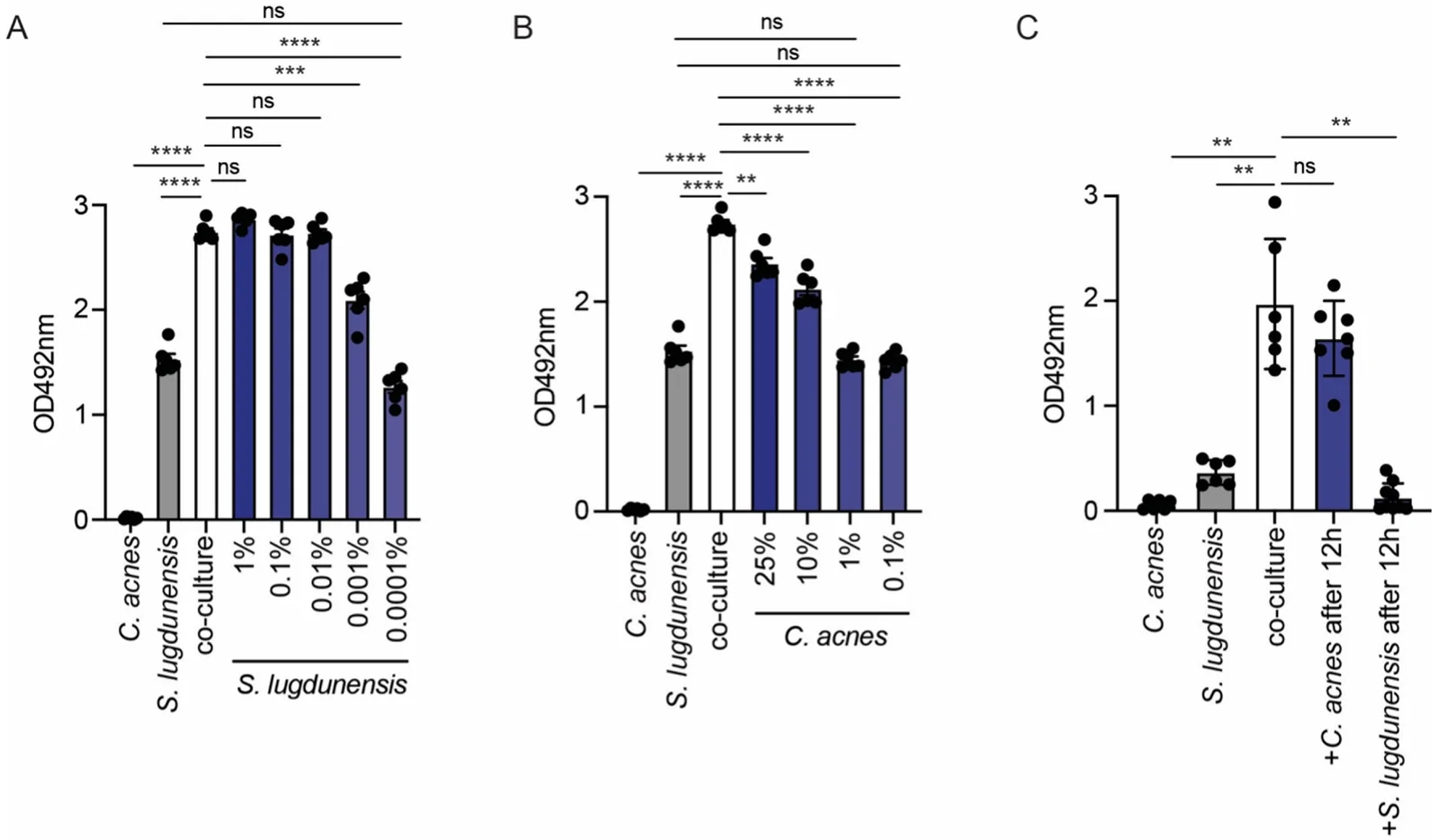
*C. acnes* and *S. lugdunensis* have differential impacts on the increased biofilm phenotype. A. Biofilm formation of co-cultures of *C. acnes* with decreasing proportions of *S. lugdunensis* after 24h of incubation. B. Biofilm formation of co-cultures of *S. lugdunensis* with decreasing proportions of *C. acnes* grown after 24h of incubation. C. *C. acnes* and *S. lugdunensis* were grown for 12h and then OD matched either *S. lugdunensis* or *C. acnes*were added and biofilm formation was measured after 12 h of exposure (total of 24h of incubation). Dots represent individual values obtained from two independent experiments and bars represent SD. Statistical analyses were performed using Brown-Forsythe and Welch ANOVA test. ** *P*<0.01, *** *P*<0.001, **** *P*<0.0001; ns: not significant.

**Figure 4:**
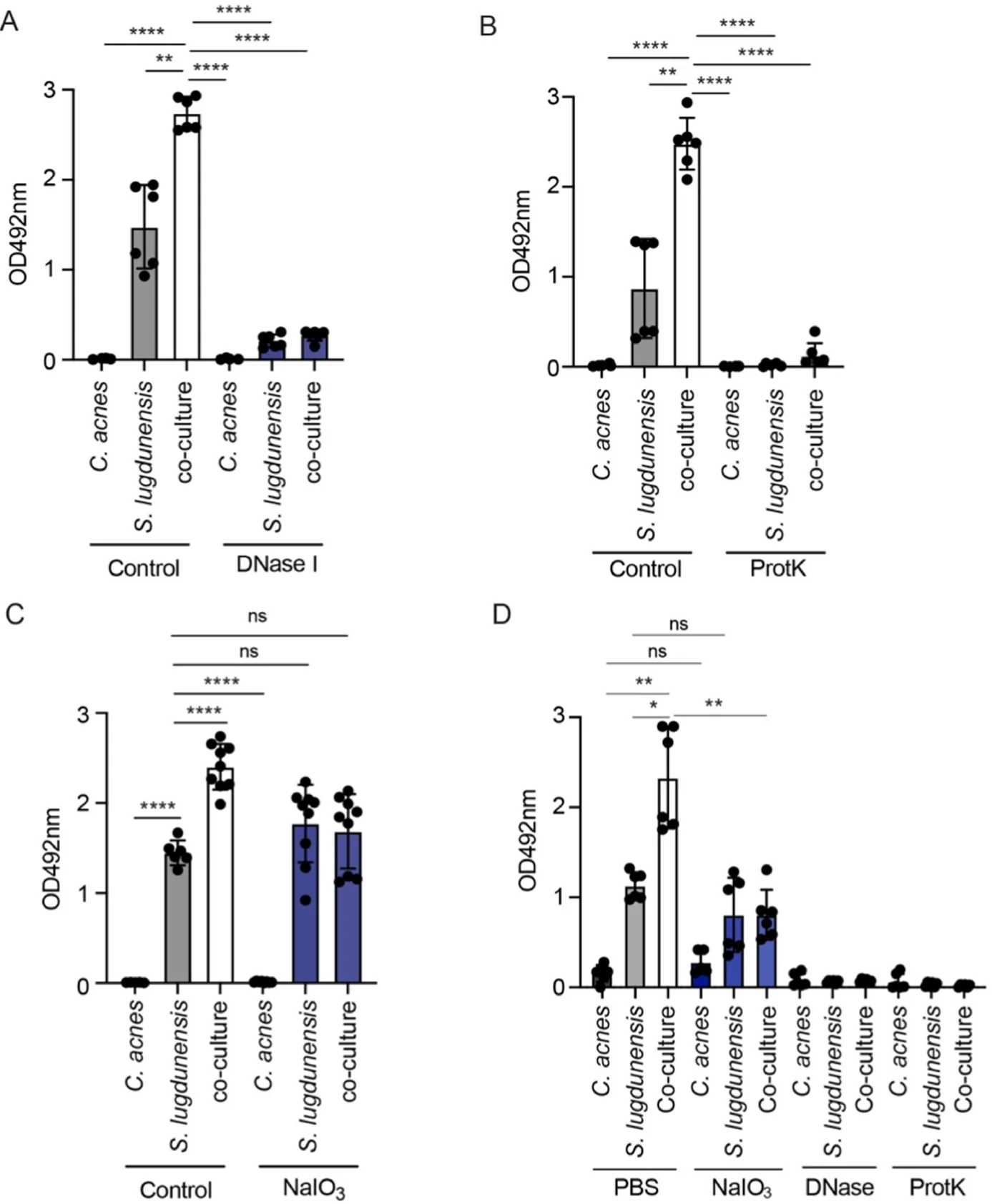
Extracellular polysaccharide production is required for the increased biofilm formation observed on the co-cultures of *C. acnes*and *S. lugdunensis*. A. Impact of DNAse I (10μg/mL) on biofilm formation of monocultures of *C. acnes, S. lugdunensis* and co-cultures after 24h of incubation. B. Impact of proteinase K (ProtK; 1.5 μg/mL) on biofilm formation of monocultures of *C. acnes, S. lugdunensis* and co-cultures after 24h of incubation. C. Impact of sodium periodate (NaIO3; 0.625 mM) on biofilm formation of monocultures of *C. acnes, S. lugdunensis* and co-cultures after 24h of incubation. D. Impact of treatment with sodium periodate (NaIO3; 40 mM), DNaseI (10μg/mL in PBS), and proteinase K (ProtK; 1 mg/mL) of 24-h biofilms of monocultures of *C. acnes, S. lugdunensis* and co-cultures. Dots represent individual values obtained from two independent experiments and bars represent SEM. Statistical analyses were performed using Brown-Forsythe and Welch ANOVA test. ** *P*<0.01, ***P<0.001; **** *P*<0.0001; ns: not significant.

**Figure 5:**
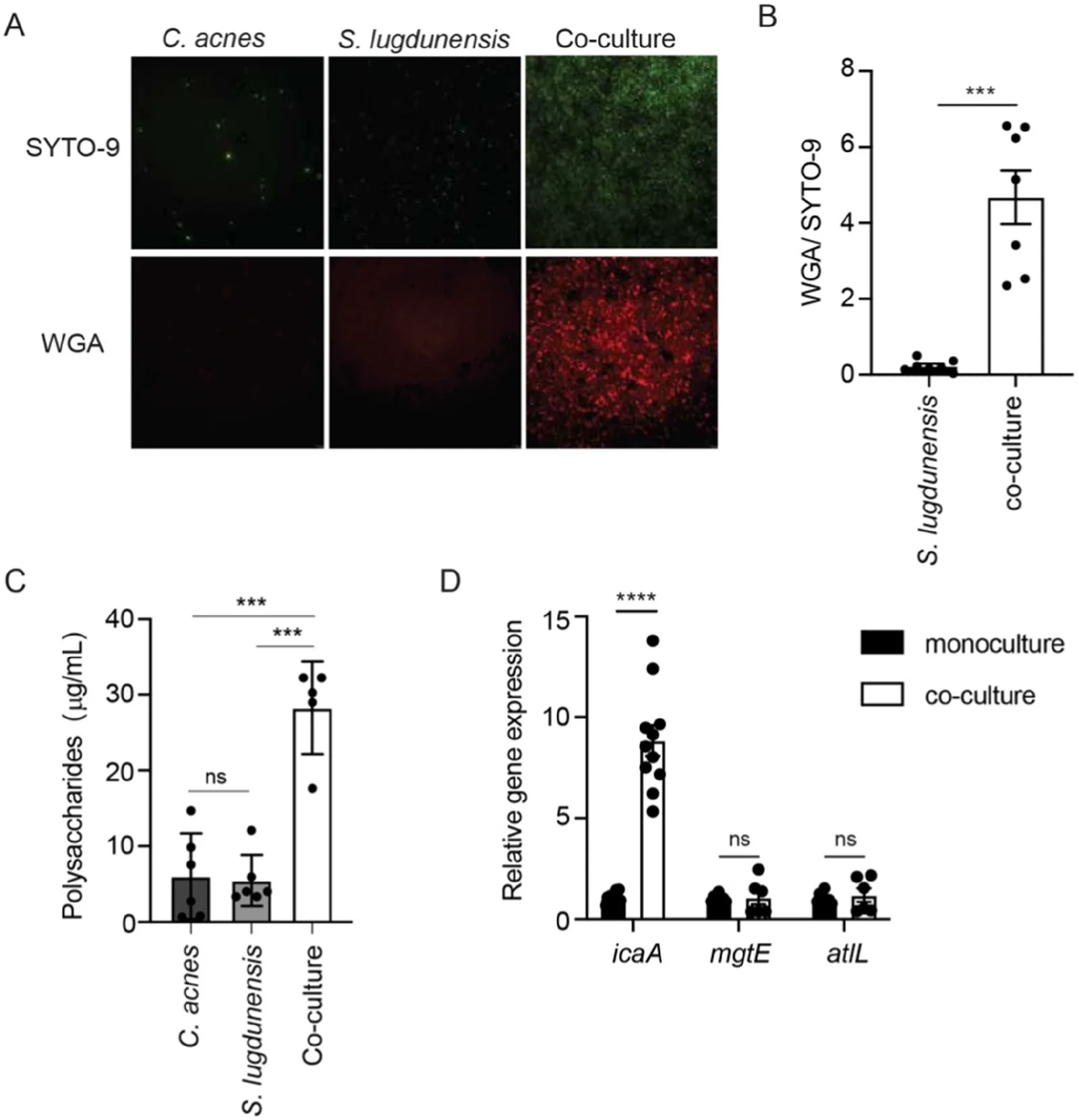
Co-culture of *C. acnes* and *S. lugdunensis*leads to increased polysaccharide production and *icaA* gene expression compared to monocultures A. Representative pictures of confocal microscopy of *C. acnes* and *S. lugdunensis* monocultures and co-cultures biofilms stained for detection of either live cells (SYTO-9) or polysaccharides (WGA). B. Detection of WGA normalized to SYTO-9 in *S. lugdunensis* monocultures and co-culture biofilms. C. Quantitative analysis of total polysaccharides extracted from biofilm matrix of *C. acnes* and *S. lugdunensis* monocultures and their co-cultures after 72h of incubation. D. Relative gene expression of *icaA*, *mgtE*and *atlL* in *S. lugdunensis* monocultures and co-cultures. Dots represent individual values obtained from two independent experiments and bars represent SEM. Statistical analyses were performed using Mann-Whitney tests (B). *** *P*<0.001; **** *P*<0.0001; ns: not significant.

## Data Availability

The datasets generated during this study are available from the corresponding author upon reasonable request.
